# Dietary Red Meat Adversely Affects Disease Severity in a Pig Model of DSS-Induced Colitis Despite Reduction in Colonic Pro-Inflammatory Gene Expression

**DOI:** 10.3390/nu12061728

**Published:** 2020-06-09

**Authors:** Tina S. Nielsen, Marlene Fredborg, Peter K. Theil, Yuan Yue, Lærke V. Bruhn, Vibeke Andersen, Stig Purup

**Affiliations:** 1 Department of Animal Science, Aarhus University, DK-8830 Tjele, Denmark; tinas.nielsen@anis.au.dk (T.S.N.); marlene.fredborg@innofond.dk (M.F.); peter.theil@anis.au.dk (P.K.T.); yuan.yue@anis.au.dk (Y.Y.); 2 Innovation Fund Denmark, DK-8000 Aarhus C, Denmark; 3 Focused Research Unit for Molecular Diagnostics and Clinical Research, IRS-Centre Soenderjylland, University Hospital of Southern Denmark, DK-6200 Aabenraa, Denmark; Laerke.Valsoe.Bruhn@rsyd.dk (L.V.B.); va@rsyd.dk (V.A.)

**Keywords:** diet, inflammatory bowel disease, porcine, dextran sulfate sodium, inflammation, histology, gene expression

## Abstract

Diet plays a substantial role in the pathogenesis and management of ulcerative colitis (UC), and epidemiologic studies indicate an association between red meat intake and increased risk of UC development. Therefore, we evaluated the effect of a red meat diet on dextran sulfate sodium (DSS)-induced colitis in pigs. Weaned pigs (42 days old) were fed either a control diet or a diet substituted with 15% minced, cooked and dried beef from experimental day 0 to 14. From day 14 to 18, half of the pigs on each diet received a daily oral dose of DSS. Dietary red meat aggravated the severity of colitis based on clinical signs of disease (negative performance score) and histopathological parameters in the colon such as erosion/ulceration and the overall inflammation score but no negative effects were observed on systemic health or small intestinal permeability. Importantly, dietary meat also caused a potential beneficial reduction in the colonic expression of the pro-inflammatory cytokines IL-17A and IL-6, the pro-inflammatory enzyme PTGS2 and in the chemokine IL-8. The present study emphasizes the potential of diet to modulate mucosal inflammation and that a red meat diet might be a risk factor for the development of inflammatory bowel disease.

## 1. Introduction

Ulcerative colitis (UC) is one of the two major chronic idiopathic inflammatory bowel diseases (IBD’s), with UC being confined to the colon [1]. Patients suffering from UC experience periods of remission interrupted by disease relapses [2,3]. The disease development is triggered by environmental factors in genetically susceptible individuals with an abnormal immune response to the commensal gut microbiota [2]. A broad spectrum of environmental factors such as use of antibiotics, diet, breast-feeding and environmental pollution has been associated with the development of IBD [4,5].

An estimated 0.3% of the population in Europe are diagnosed with IBD [6] and although the incidence rate is stabilizing in the Western world, the increasing global prevalence of IBD [7] will put a continuous pressure on the health care system in the affected countries [8]. The IBD incidence rate seems to rise concurrently with Westernization in Asia [7] making IBD a global health issue. A dietary pattern associated with high intakes of meat has been linked to increased risk of IBD in children [9] and UC patients have reported that consumption of meat worsens their symptoms [10]. Some human studies report significant associations between meat intake and increased risk of UC development [11,12] and relapse [13], whereas others do not [14]. A meta-analysis of seven epidemiological studies showed a significant association between meat intake (red meat in particular) and risk of UC [15].

One of the proposed mechanisms for the negative effect of meat on IBD development is that a high meat intake may increase the amount of proteins, peptides and amino acids reaching the colon where they are metabolized by gut bacteria [16]. Protein fermentation results in the formation of a variety of metabolites that may cause intestinal dysfunction and render the intestinal mucus layer penetrable to, for example, bacteria, by reducing the di-sulfide bonds in the mucus network [16,17,18]. Thus, microbes may reach the epithelium and activate the immune system [19]. However, a clear link between meat intake and risk of IBD development or disease relapses has yet to be confirmed [5,20].

The use of animal models provide a way to study the influence of dietary factors on IBD pathogenesis in a controllable environment [21] and rodent models of dextran sulfate sodium (DSS)-induced colitis are frequently used to study the pathogenesis of UC [22]. However, pigs may be an even better model system since humans and pigs share several traits, such as comparable nutrient requirement, analogous metabolic processes, corresponding immunology and exhibit anatomically and physiologically similar gastrointestinal tracts [23,24,25,26,27,28]. Colitis has therefore been induced by DSS in typically very young piglets (3–15 days old) to study preventive or therapeutic effects of, for example, amino acid supplementation, fatty acids, mannanase-hydrolyzed copra meal and soy-derived di- and tripeptides [29,30,31,32,33].

In the present study, we examined the effect of red meat intake prior and during DSS treatment on DSS-induced colitis symptoms and on systemic and local immune response parameters using a porcine model of experimental UC. The pigs applied in the present study were older (42 days of age) than pigs in previous DSS-model studies [29,30,31,32,34] and their gastrointestinal tracts thereby more mature to better mimic the adult phase of human life where IBD disease onset is at its peak [17]. We hypothesized that dietary meat would aggravate DSS-induced colitis development.

## 2. Materials and Methods

The care and housing of animals used in the experiment complied with Danish laws and regulations for the humane care and use of animals in research (The Danish Ministry of Justice, Animal Testing Act no. 1306 of 23 November 2007) and performed under the license obtained from the Danish Animal Experimentation Inspectorate, Ministry of Food, Agriculture and Fisheries. The health and wellbeing of the animals was monitored daily and immediate action was taken to alleviate suffering caused by the experimental treatments.

### 2.1. Animals and Experimental Design

Twenty-four 6-week-old castrated male pigs (cross-bred Yorkshire, Landrace, Duroc) weaned on day 28 (9.9 kg ± 0.1) were obtained from a commercial swine herd. To be included in the study pigs should not previously have been treated with antibiotics. The experiment was conducted in three blocks of eight pigs per block. The pigs in each block originated from two litters (four per litter) and were housed in pairs (one from each litter) in pens with concrete floor and access to water ad libitum and some manipulative material (sawdust). During the first two experimental weeks, pigs received one of two dietary treatments. One group was fed a standard pelleted diet (Prime Midi Piller U; DLG, Randers, Denmark) without addition of zinc oxide ad libitum twice daily (8 a.m. and 3 p.m.). The other group of pigs were offered ~15% (by weight) of the pelleted feed intake the previous day as minced, cooked, dried (80 °C for 24 h) beef (Hørkram Foodservice, Hørning, Denmark) before the morning feeding followed by the pelleted diet ad libitum. In the afternoon, pigs were offered any leftover meat from the morning feeding before the pelleted diet *ad libitum*. The two pigs housed in a pen received the same dietary treatment and were separated during feeding. Leftover meat was weighed for each individual pig after the afternoon feeding, where upon leftover-pelleted feed was registered before the morning feeding to calculate feed intake on the pen level (two pigs) and meat intake on an individual level.

**Figure 1:**
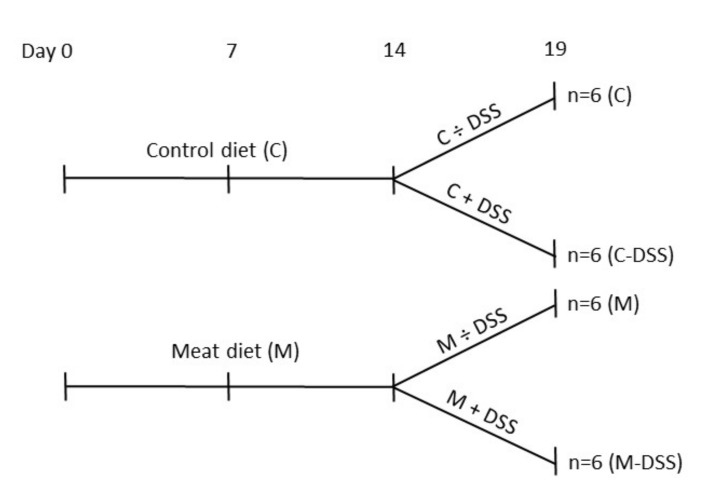
Overview of time-flow of the study and the four experimental treatments from day 14 to 18 (slaughter day 19); control diet (C), control diet + dextran sulfate sodium (DSS) (C-DSS), meat diet (M) and meat diet + DSS (M-DSS). Pigs were 42 days of age at experimental day 0.

By the end of the second week (eight weeks of age), pigs receiving the standard diet (Control; C) or the standard diet + meat (M) were divided into daily treatments of 20 mL sterile saline solution (0.9% NaCl) + 5 mL apple juice with or without 1.25 g DSS/kg BW (DSS; dextran sulfate sodium salt, MW 36–50 kDa, MP Biomedicals, Santa Ana, CA, USA). The experimental set-up is shown in Figure 1. The solution was administered orally by a syringe fitted with an oral rubber probe. The dose of DSS was determined from a dose–response pilot study (0, 0.625, 1.25 or 2.5 g DSS/kg BW) (unpublished data) and from previous reports of DSS-induced porcine colitis models [29,31,34]. Pigs received the treatment each morning before the feeding for five days (Sunday to Thursday; day 14 to 18) after fasting from the evening before. Body weight was recorded every morning to calculate the amount of DSS to be administrated.

### 2.2. Registrations and Samples

The health and wellbeing of animals were monitored and scored on day 14, 16, 18 and before slaughter at day 19 according to the criteria described in Table 1. The fecal texture score 0, 2 and 4 in Table 1 corresponded to score 1, 2–3 and 4, respectively, on the descriptive classification scale for clinical assessment of fecal consistency in pigs described by Pedersen and Toft [35]. The score 0, 2 or 4 for blood in feces and a pig’s overall condition (Table 1) was designated by experienced stable personnel used to monitor the health and wellbeing of pigs. The individual scores on each day for BW loss, fecal texture, presence of blood in the feces and the overall condition of the animal were summarized into a negative performance score (up to a maximum of 16). A higher negative performance score is therefore indicative of an animal being more negatively affected overall.

Fasting jugular blood was obtained on day 0, 7, 14, 16, 18 and before slaughter day at 19, centrifuged (3200 rpm, 12 min, 4 °C) and plasma stored (−20 °C) until analyzed. On day 19, pigs were anesthetized (Zoletil 50, 1 mL/10 kg BW), injected with sodium pentobarbital and bled from the jugular vein. The positions at 10%, 50%, and 90% of the length of the small intestine (Si) (descending) were termed Si10, Si50 and Si90, respectively, and similarly the positions at 25%, 50% and 75% of the length of the colon (Co) (descending) were termed Co25, Co50 and Co75, respectively. A macroscopic score of the Si10, Si50, and Si90, the cecum and Co25, Co50, and Co75 was performed using the following assessment criteria: 0 = normal tissue, 1 = reddish inflamed, 2 = erosion, and 3 = ulceration. In addition, a score of 1 was assigned if there were other notable differences in each segment. The scores for each animal were summed into a total score (colitis score).

Samples of clean tissue from Co25, Co50, and Co75 were obtained and placed in RNA-later at 5 °C for 24 h before storage at −80 °C for later isolation of colonic RNA and analysis of gene expression. Scraps of the intestinal mucosa were obtained with a cover glass slide from the clean midsection of the small intestine (Si50) and the colon (Co50) and stored at −20 °C until IgA analysis.

### 2.3. In Vivo Intestinal Permeability Test

Fasting pigs were subjected to a gut permeability test with D-mannitol on day 19 before slaughter, as previously described [34]. Briefly, D-mannitol (0.6 g/kg BW) was dissolved in 10 mL saline solution (0.9% NaCl) and administrated orally by a syringe fitted with an oral rubber probe. Jugular blood was obtained in heparinized tubes at 0, 35, and 70 min and plasma was immediately collected by centrifugation at 800× *g* for 5 min. D-mannitol concentrations was determined by an enzymatic-fluorometric method, principally analogue to determination of glutamic acid [36]. However, in this instance, mannitol dehydrogenase (EC 1.1.1.67) was used to start the reaction. Concentrations were calculated from a D-mannitol standard curve.

### 2.4. Plasma and Mucosal Analysis

Haptoglobin was determined chemically due to its ability to bind to hemoglobin, Phase™, Tridelta Developments, Wicklow, Ireland. All analyses were performed using an autoanalyzer, ADVIA 1800^®^ Chemistry System (Siemens Medical Solutions, Tarrytown, NY, USA). The intra assay variabilities were in all instances below 2% (CV); inter assay variations were in all instances below 4.5% (CV). Pig C-reactive protein (CRP) was analyzed by a commercial ELISA assay (Tridelta Developments Ltd., Maynooth Co. Kildare, Ireland). Manufacturer’s instructions were followed. Intra- and inter assay variations were below 8% (CV).

The concentrations of total IgG, IgA, and IgM in plasma and IgA in mucosal scrapings of Si50 and Co50 were measured using the pig Ig ELISA quantification kit (Bethyl Laboratories, Montgomery, TX, USA). Prior to the assay of IgA in mucosa scrapings were vortexed in PBS (1:10, wt/wt), centrifuged at 2000× *g* for 10 min, and the supernatant was collected for analysis. The concentration of IgA in mucosa was expressed relative to total protein, which was quantified by the Auto analyzer Advia 1650 (Siemens Medical Solutions, Tarrytown). All Ig assays were performed according to the manufacturer’s instructions and run in duplicates. The intra-assay (within-plate) CV for the ELISA was 0.4–5.8% and the inter-assay (between-plate) CV was 0.6–5.9%.

### 2.5. Histopathology

Tissue samples of Co25, Co50 and Co75 were fixed in 10% formalin, dehydrated in graded ethanol series and embedded in paraffin. Samples were cut into 5-μm thick sections and stained with hematoxylin and eosin (H&E). Histological inflammation was evaluated based on cryptitis (presence of neutrophils in epithelia = 1, none = 0), crypt abscesses (presence of neutrophils in lumen = 1, none = 0), ulceration (presence = 1, none = 0) and an overall inflammation score (0 = none, 1 = slight, 2 = moderate, 3 = severe). The cross sections were assessed in a blinded manner.

### 2.6. Colonic Gene Expression (qRT-PCR)

Total RNA was extracted from intestinal tissue of the Co25, Co50 and Co75 using the NucleoSpin II kit (Macherey-Nagel GmbH & Co. KG., Düren, Germany) according to the manufacturer´s instructions. RNA purity and concentration were examined by measuring absorbance at 260–280 nm (NanoDrop ND-8000 UV-vis spectrophotometer, NanoDrop Technologies, Wilmington, DE, USA). Purified RNA was reverse-transcribed with oligo-dT and random primers and Superscript III RNase H reverse transcriptase kit (Invitrogen, Taastrup, Denmark) according to the manufacturer’s protocol. Absence of amplification of genomic DNA was tested using porcine DNA for the quantitative reverse transcription PCR analyses. Reverse-transcribed material was amplified and quantified with TaqMan Universal PCR Master Mix or Power SYBR Green PCR Master Mix (Applied Biosystems, Stockholm, Sweden) depending on the analyzed gene on an ViiA7 (LifeTechnologies, Taastrup, Denmark) using 384-well plates with 10 µL reaction volume. Intestinal mucosal scrapings (Co25, Co50 or Co75) were analyzed for expression of 8 selected gene transcripts, using pig-specific primers and probes (Supplementary Table S1). Two reference genes were tested (GAPDH and β-actin) but only β-actin was found suitable as endogenous control. The raw gene-expression data was obtained as Ct values (cycle number at which logarithmic plots cross a calculated threshold) according to the manufacturer’s guidelines, and used to determine ΔCt values (ΔCt = Ct of the target gene − mean Ct of the housekeeping gene), which were used for the statistical analyses. Then, ΔΔCt =ΔCt for e.g., the meat diet (M)—ΔCt for the weaner diet (C) was calculated and the relative gene-expression was derived using (1 + efficiencies) ^−ΔΔCT^ method and the fold change (FC) was reported.

### 2.7. Statistical Analysis

The statistical analysis applied was performed in the Mixed procedure of SAS (SAS institute, Inc., Cary, NC, USA). Data on immunological parameters in blood, negative performance score, gut permeability, gene expression and colonic histology were analyzed using the following overall model:

X*_ijklmn_* = µ + α*_i_* + β*_j_* + αβ*_ij_* + γ*_k_* + βγ*_jk_* + αβγ*_ijk_* + δ*_l_* + δη*_lm_* + ν*_ijln_* + ε*_ijklmn_* (1)

were α*_i_* is diet (*i* = C or M); β*_j_* is treatment (*j* = with or without DSS); αβ*_ij_* is the interaction between diet and DSS treatment; γ*_k_* is the day (*k* = 0, 8, 14, 16, 18 or 19 for immunological parameters; *k* = 14, 16, 18 or 19 for negative performance score), time-point (*k* = 0, 35 or 70 min) or segment of the colon (*k* = Co25, Co50 or Co75); βγ*_jk_* is the interaction between DSS treatment and day (or DSS treatment × time-point or DSS treatment × segment); αβγ*_ijk_* is the interaction between diet, DSS treatment and day (or diet × DSS treatment × time-point or diet × DSS treatment × segment); δ*_l_* is the effect of block (*l* = 1, 2 or 3); δη*_lm_* is the random component related to the interaction between block and litter (*m* = 1, 2, …, 12) and ν*_ijln_* is the random component related to the pig (*n* = 1, 2, …, 24). Pig and the interaction between block and litter were included as random components to account for repeated measurements within a pig (for certain endpoints) and within the litter. The covariance structure for repeated measures within pigs and the interaction between block and litter was modelled using variance component option and the residual error component is defined as ε*_ijklmn_*. For data on colonic gene expression, αβγ*_ijk_* was omitted from the model and BW d14 within block was included as a covariate as this was the time point for +/− DSS treatment. Levels of significance were reported as being significant when *p* ≤ 0.05. The random effects and residuals were assumed to be normally distributed and independent and their expectations were assumed to be zero. Data on BW d14, d19, BW gain, feed and meat intake were analyzed by the same statistical model and under the same assumptions, except that γ*_k_*, βγ*_jk_*, αβγ*_ijk_* and ν*_ijln_* were omitted from the model, and BW day 0 within block was included as a covariate in the analysis of BW day 14 (BW d14 within block was included as a covariate in the analysis of BW at slaughter d19).

## 3. Results

### 3.1. Body Weight, Feed Intake and Negative Performance Score

One pig receiving the control diet and DSS (C-DSS) treatment was euthanized at day 15 due to illness. On day 14 before DSS treatment was initiated, meat-fed pigs showed a higher BW compared with control-pigs (LSmean ± SE 16.3 ± 0.6 vs. 14.9 ± 0.6 kg, respectively, *p* = 0.01); a difference in BW that was still apparent at slaughter d19 (18.4 ± 0.6 vs. 16.5 ± 0.6 kg for meat and control-fed pigs, respectively, *p* = 0.01, Table 2). From day 0 to 13, meat-fed pigs consumed 55–68 g/d of meat and they showed a tendency (*p* = 0.08) for a lower intake of the pelleted diet (Table 2). DSS treatment reduced the daily feed intake from day 14 to 18 by 23% (*p =* 0.05) and the daily weight gain by 36% compared with non-DSS treated pigs (*p* = 0.03) irrespective of diet (Table 2).

**Figure 2:**
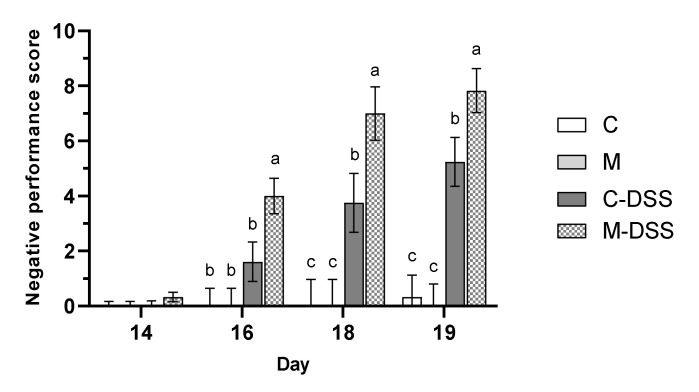
Negative performance score on day 14 to 19 for pigs receiving the control diet (C), the meat diet (M), the control diet + DSS (C-DSS) or the meat diet + DSS (M-DSS) (*n* = 6/group, except C-DSS *n* = 5). Data are presented as least square (LS) means ± SEM. Different letters (a, b, c) indicate a significant effect of treatment within day (*p* ≤ 0.05).

The health and wellbeing of the pigs was scored (see Table 1 for criteria) at day 14, 16, 18 and 19 and expressed as a negative performance score (Figure 2) where a higher score is indicative of being more negatively affected by the treatment.

Overall, DSS treatment resulted in a significantly higher negative performance score, than in pigs not receiving DSS (*p* < 0.001). However, the meat diet in combination with DSS treatment resulted in the highest negative performance score from day 16 and onwards indicating that those pigs overall were more affected in terms of weight loss relative to the day before, fecal consistency, blood in the feces and on their overall condition compared with the other groups.

### 3.2. In Vivo Gut Permeability

**Figure 3:**
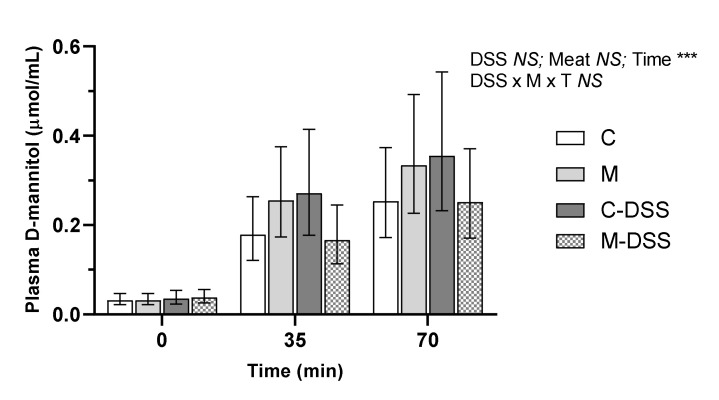
Effect of red meat supplementation and DSS treatment on in vivo gut permeability. Values represent LS means ± 95% confidence limits due to logarithmic transformation of data. Control diet, C (*n* = 6), meat diet, M (*n* = 6), control diet + DSS, C-DSS (*n* = 5), meat diet + DSS, M-DSS (*n* = 6). *NS* = non-significant, *** *p* < 0.001.

D-mannitol was used to measure the in vivo intestinal permeability on the day of slaughter in pigs receiving the four different treatments. There were no treatment-induced differences in plasma D-mannitol concentrations within a time point (Figure 3). The relationship between plasma D-mannitol concentrations and time post D-mannitol infusion was linear across all groups, but there was no difference in the slope of the linear relationships (y = 0.003x + 0.05; y = 0.004x + 0.07; y = 0.005x + 0.08 and y = 0.003x + 0.05 for the C, M, C-DSS and meat diet + DSS (M-DSS) groups, respectively; *p* = 0.48).

### 3.3. Macroscopic and Histopathological Evaluation

The gross appearance of Si10, Si50 and Si90, the cecum and Co25, Co50 and Co75 were macroscopically scored during necropsy and a “colitis score” for each animal was calculated. The scores for the three small intestinal segments were part of the overall colitis score but no differences in small intestinal segments colitis score between treatments were observed (data not shown). DSS treatment increased the colitis score to 3.4 ± 0.5 compared to 1.0 ± 0.4 for untreated pigs (*p* < 0.01), whereas diet and the diet×DSS-treatment interaction showed no significant effect (*p* = 0.37 and *p* = 0.94, respectively).

The severity of colonic inflammation was also evaluated by examining H&E-stained colon sections. The assessment included the percentage of pigs with cryptitis (neutrophils in epithelia), crypt abscesses (neutrophils in lumen), erosion/ulceration and an overall inflammation score (Table 3). DSS treatment caused a higher percentage of pigs with ulceration (*p* = 0.03) and a higher overall inflammation score in the proximal colon (Co25) (*p* < 0.01), irrespective of diet. DSS treatment also caused a higher percentage of pigs with ulceration and a higher overall inflammation score in the mid (Co50) and distal colon (Co75) (*p* < 0.001) compared to untreated pigs. However, compared to DSS treatment alone, the M-DSS treatment further increased the percentage of pigs with ulceration and the overall inflammation score in the mid and distal colon (Table 3).

In both mid and distal colon the number of pigs showing ulceration more than doubled (from 40 to 83%), whereas the total inflammation score in both segments was 1.4–1.5 times higher following the M-DSS compared with the C-DSS treatment. In the distal colon, DSS treatment also increased the percentage of pigs with crypt abscesses (*p* < 0.01) and the percentage of pigs with cryptitis (*p* = 0.06).

### 3.4. Colonic Gene Expression

**Figure 4:**
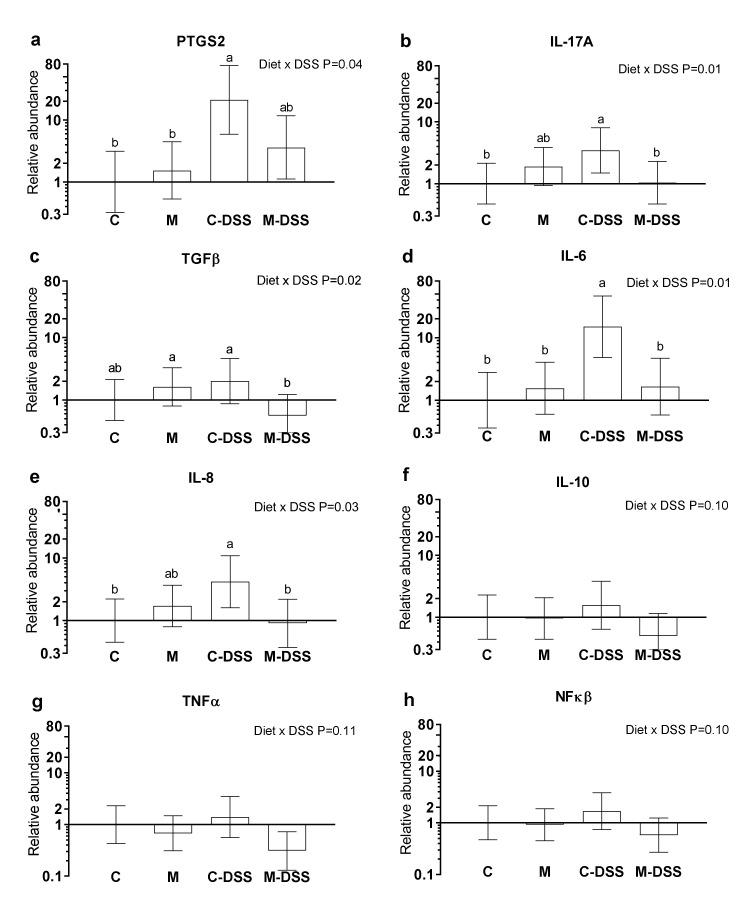
Relative mRNA abundance in the colon of pigs fed the control diet (C), the meat diet (M), the control diet + DSS (C-DSS) or the meat diet + DSS (M-DSS) (*n* = 6/group except C-DSS *n* = 5). (**a**) PTGS-2; (**b**) IL-17A; (**c**) TGFβ; (**d**) IL-6; (**e**) IL-8; (**f**) IL-10; (**g**) TNFα; (**h**) NFκβ. Data are presented as the means ± 95% confidence intervals relative to the abundance in the control-treated pigs (relative expression = 1.00). Note that the scale on the *Y*-axis is logarithmic and that the units are arbitrary. Statistically significant (*p* ≤ 0.05) effect of treatment is indicated with different letters (**a**,**b**).

Prostaglandin-endoperoxide synthase 2 (PTGS2) also known as cyclooxygenase-2 (COX-2), interleukin 17A (IL-17A), transforming growth factor-beta (TGFβ), interleukin 6 (IL-6), interleukin 8 (IL-8), interleukin 10 (IL-10), tumor necrosis factor-alpha (TNFα) and nuclear transcription factor-kappa-beta (NFκβ) mRNA abundances are shown in Figure 4a–h. There was no effect of segment (Co25, Co50 or Co75) on the analyzed genes (*p >* 0.05) and therefore results are presented in Figure 4 across colon segments. DSS treatment combined with the control diet increased PTGS2, IL-17A, IL-6 and IL-8 expression 21-fold, 3-fold, 15-fold and 4-fold, respectively, compared with untreated pigs (C) (Figure 4a,b,d,e). Common for these genes were that the meat diet (M-DSS treatment) restored expression levels towards the control animals (although not statistically significant for PTSG2). Additionally, for TGFβ, the M-DSS treatment lowered the relative mRNA abundance level compared with the C-DSS group, C-pigs being intermediate. For IL-10, TNFα and NFκβ there was a statistical tendency (*p* = 0.10–0.11, Figure 4 f–g) for an interaction between diet and DSS treatment and thereby for the meat diet to exhibit a similar reducing effect on DSS-induced expression of these genes.

### 3.5. Immune Response Parameters in Blood and Intestinal Mucosa

**Figure 5:**
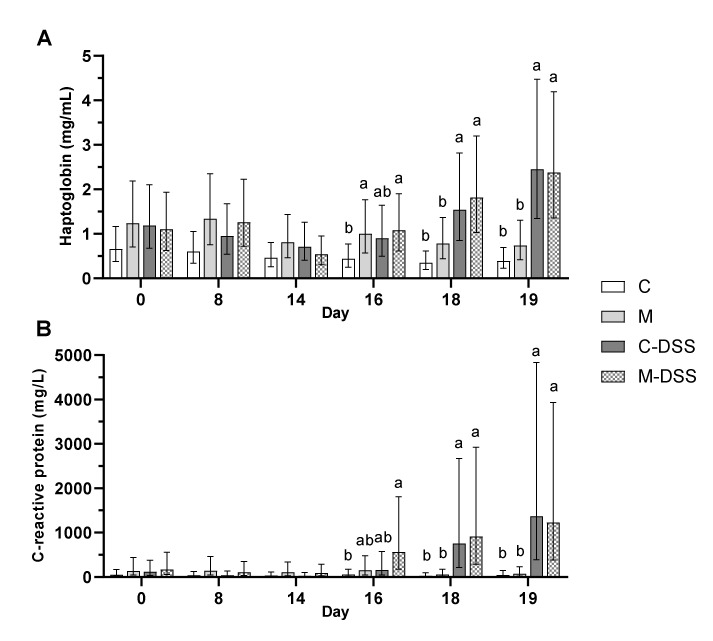
Haptoglobin (**A**) and C-reactive protein (CRP; **B**) in plasma of pigs receiving the four different treatments from day 14 to 18 (*n* = 6/group, except C-DSS *n* = 5). Data are presented as LS means ± 95% confidence intervals due to logarithmic transformation of data. Different letters indicate a significant effect of treatment within day (*p* ≤ 0.05).

The concentrations of circulating and intestinal mucosal immune response parameters were evaluated as measures of systemic and local inflammation. The concentration of haptoglobin in plasma was almost two times higher (1.7 mg/mL) at day 18 and more than three times higher (2.4 mg/mL) at day 19 following DSS treatment (starting day 14) compared with untreated pigs (0.6 and 0.6 mg/L for day 18 and 19, respectively) irrespective of diet (Figure 5A). DSS treatment showed a similar effect on C-reactive protein (CRP) in plasma. On day 18, the concentration of CRP was more than 19 times higher (835 mg/L) in DSS-treated pigs compared with untreated pigs (43 mg/L) and on day 19 the difference was an almost 22 times higher CRP concentration in DSS-pigs (1300 vs. 60 mg/L in untreated pigs) irrespective of diet (Figure 5B).

There was no statistically significant differences in levels of immunoglobulins (IgG, IgA and IgM) between treatment groups in blood samples obtained day 0, 8, 14, 16, 18 and 19 (data not shown). DSS treatment increased levels of IgA in the mucosa of the mid colon (Co50), (average 15.6 mg/g protein for the C-DSS and M-DSS group) relative to untreated pigs (average 5.5 mg/g protein for the C and M group) (*p* < 0.01) but there was no effect of diet (*p* = 0.81) and no interaction between diet and DSS treatment (*p* = 0.98). Mucosal IgA in the small intestine (Si50) was unaffected by diet and DSS treatment (data not shown).

## 4. Discussion

The purpose of this experiment was to determine if a diet with red meat would aggravate disease symptoms and inflammation associated with experimental colitis in pigs. Diet composition plays a substantial role in the pathogenesis and management of colitis [37], and epidemiologic studies indicate that dietary red meat may be a negative factor in relation to UC [15]. No human dietary intervention studies have investigated such a relationship but the impact of red and processed meat on risk of late-onset chronic inflammatory disease including IBD is currently being evaluated in a large prospective cohort study in Denmark [17]. Although the exact pathophysiological mechanisms in which diet plays a role in IBD development remain unknown, several explanations including its effects on composition of gut microbiota, production of microbial metabolites, alterations in mucosal immunity, and mucosal barrier function have been put forward [38]. Dietary red meat intake may increase the amount of proteins, peptides and amino acids reaching the colon where they can be metabolized by gut bacteria resulting in formation of branched-chain fatty acids (BCFA), ammonia, phenolic and indolic compounds, biogenic amines, hydrogen sulfide and nitric oxide [16]. A proposed mechanism for a relationship between IBD risk and/or development and red meat intake involves some of these compounds that may induce breakdown of the colonic mucus barrier function so that microbes and toxins may reach the epithelium and activate the immune system [16,17,18,19].

Our results show that compared with untreated pigs, DSS reduced the BW gain, increased the negative performance score of pigs, increased the colitis score at slaughter, and most histopathological parameters of inflammation (cryptitis, ulceration and inflammation score), especially in the mid and distal colon. Furthermore, DSS increased the level of circulating inflammation markers haptoglobin and CRP, as well as IgA locally in the colon. Overall, these DSS-induced effects are well in accordance with previous results from pig [29,30,31,32,33,34,39] and rodent studies [40,41]. Additionally, in accordance with previous results from a study of DSS-induced colitis in piglets weaned at day 21 [42], we found that DSS treatment caused an increase in the expression of the pro-inflammatory cytokines IL-17A and IL-6, the pro-inflammatory enzyme PTGS2 and the chemokine IL-8 in the colon; expression of the latter is shown to be significantly higher in inflamed versus normal colonic mucosa of IBD patients [43].

However, we did not observe a DSS-induced increase in gut permeability assessed by using D-mannitol as a non-metabolizable monomer marker of small intestine-specific permeability [34,44], although this has been reported in previous pig studies [29,30,34]. In general, the pigs used in the mentioned studies were much younger (3–5 days old) than the pigs applied in our study (8 weeks old) when DSS treatment was initiated. Our results suggest that small intestinal permeability in older pigs 8 weeks of age was not negatively affected by either DSS treatment or dietary red meat. Moreover, we did not observe any negative effects of DSS treatment on the gross appearance at necropsy of the proximal, mid or distal small intestine (Si10, Si50 and Si90) and no effect on small intestinal mucosal IgA concentration, together indicating that the small intestine was relatively unaffected by DSS treatment.

Feeding the red meat prior to and during DSS treatment caused the negative pig performance score (a summarized score of BW loss, fecal texture, presence of blood in the feces and the overall condition of the animal) to be higher than when DSS was given with the control diet, i.e., meat worsened the effect of the DSS treatment on these indicators of disease severity. The meat diet also aggravated the effect of DSS treatment measured as the percentage of pigs exhibiting ulceration and on the inflammation score, especially in the mid and distal colon. To our knowledge, there are no previous studies on the effects of dietary red meat in IBD-model systems. However, Schepens et al. [45] studied the effect of dietary heme (the iron-containing porphyrin pigment of red meat), mimicking red meat on trinitrobenzene sulfonic acid (TNBS)-induced colitis in rats. Rats were fed a high-fat control diet or a similar diet supplemented with 0.5 mmol heme per kg of diet. This heme concentration corresponds to a meat intake of 500 g per day [45]; our meat-fed pigs consumed 55–87 g meat per day depending on the experimental day. Colitis severity was evaluated and several inflammation markers quantified in colonic mucosa after colitis induction. The combination of dietary heme and TNBS treatment resulted in more diarrhea and a higher cytotoxicity of the fecal water than in control fed TNBS treated animals. Body weight re-gain after TNBS infusion was also less and systemic health (increased acute-phase protein AGP) during colitis was worse in heme-fed animals compared with controls. Overall, heme-induced luminal stress aggravated the severity of colitis, despite an increase in colonic heat-shock protein (HSP) expression [45]. Inducible HSP are produced in response to several kinds of stress and serve to stabilize other proteins and preserve their function [46] and therefore are potentially a beneficial event. Interestingly, Schepens et al. [45] found that the concentration of the pro-inflammatory markers MPO (myeloperoxidase) and IL-1β locally in the colonic mucosa was reduced following dietary heme. This corresponds with our finding, that the meat diet combined with DSS treatment reduced the expression of the pro-inflammatory cytokines IL-17A and IL-6, the pro-inflammatory enzyme PTGS2 and the chemokine IL-8 in the colon compared to control pigs treated with DSS.

The discrepancy between the effect of dietary red meat on colonic expression of certain inflammatory markers, on the one hand, and clinical health, determined as the negative performance score as well as colonic inflammation, determined histologically as erosion/ulceration and inflammation score, is interesting. Pigs in the M-DSS group appeared to be more severely negatively affected (measured as the negative performance score, Figure 2) by DSS at a faster rate during the five-day DSS-exposure period compared with the C-DSS pigs. The M-DSS pigs may therefore have been at a more progressed disease stage or earlier restitution stage than the C-DSS pigs at the time of slaughter where colonic tissue for gene expression was obtained. However, for this to be part of an explanation we would expect a higher expression of TGF-β as a primary mediator of mucosal repair and IL-10 as a secondary factor in the M-DSS group, which was not the case. It is widely accepted that TGF-β has an important function in regulation of inflammation and tissue repair and it has been suggested that IL-10 is a necessary secondary factor that facilitates TGF-β production [21].

Overall, the results of our study should be interpreted with the relatively low number of animals (*n*= 5 or 6) per treatment in mind. Additional analysis that could also potentially contribute to a better understanding of the observed dual effect of the meat diet includes microbial analysis (α-diversity, number of sulfate-reducing bacteria) and concentration of biogenic amines in colonic digesta. The intestinal microbiota in IBD patients have been shown to be different from healthy subjects [47] and a reduction in species richness (α-diversity) in IBD patients has been reported [48]. Furthermore, an increase in sulfate-reducing bacteria and genes encoding for metabolism of the sulfur-containing amino acid cysteine, has been reported in IBD patients [49].

## 5. Conclusions

The present study shows that dietary red meat aggravated the severity of colitis based on clinical signs of disease (negative performance score) and histopathological parameters in the colon (erosion/ulceration and the overall inflammation score) but no negative effects were observed on systemic health or small intestinal permeability. Importantly, dietary meat also caused a potential beneficial reduction in the colonic expression of the pro-inflammatory cytokines IL-17A and IL-6, in the pro-inflammatory enzyme PTGS2 and in the chemokine IL-8. We hypothesize that the faster disease progression and severity during the 5-day experimental period among the DSS treated meat-fed pigs may contribute to their unexpected favorable colonic gene expression. The present results emphasize the potential of diet to modulate mucosal inflammation and the study implies that a diet high in red meat might be a risk factor for the development of IBD. However, more studies are necessary to confirm these findings.

## Figures and Tables

**Table 1:** Criteria for monitoring the health and wellbeing of the pigs.

Weight Loss Rel. to the Day before	Fecal Texture	Blood in the Feces	Overall Condition
0 = None	0 = normal	0 = normal	0 = normal
1 = 1–5%	2 = thin/soft	2 = little-moderate	2 = slightly affected
2 = >5–10%	4 = diarrhea	4 = moderate-heavy	4 = highly affected
3 = >10–15%			
4 = >15%			

**Table 2:** Growth performance and feed/meat intake for pigs receiving the four different treatments. Least square mean values with their standard errors, *n* = 6/group except C-DSS *n* = 5.

	Treatment					*p*-Value		
	C	M	C-DSS	M-DSS	SEM	Diet	DSS	D × DSS
BW d14, kg	14.7 ^b^	15.7 ^ab^	15.1 ^b^	16.9 ^a^	0.71	0.01	0.13	0.38
BW d19, kg	16.9 ^ab^	18.1 ^a^	16.2 ^b^	18.8 ^a^	0.80	0.01	0.99	0.35
BW gain d14-18, g/day	426 ^a^	481 ^a^	215 ^b^	366 ^ab^	74	0.12	0.03	0.50
Feed intake d0-13, g/day	502	488	518	428	30	0.08	0.60	0.19
Meat intake d0-13, g/day	0 ^c^	55 ^b^	0 ^c^	68 ^a^	3	<0.001	0.02	0.01
Feed intake d14-18, g/day	443 ^ab^	550 ^a^	366 ^b^	401 ^ab^	53	0.21	0.05	0.52
Meat intake d14-18, g/day	0 ^c^	66 ^b^	0 ^c^	87 ^a^	9	<0.001	0.23	0.22

^a,b,c^ Means within a row with unlike superscript letters were significantly different (*p* ≤ 0.05).

**Table 3:** Histopathological parameters measured as percentage of pigs with cryptitis, crypt abscesses, erosion/ulceration and the overall inflammation score in the proximal (Co25), mid (Co50) or distal (Co75) colon after five days of DSS or placebo treatment in pigs fed the control (C) or the meat (M) diet (*n* = 6/group, except C-DSS *n* = 5). Least square mean values with their standard errors.

	Treatment					*p*-Value		
	C	M	C-DSS	M-DSS	SEM	Diet	DSS	D × DSS
Co25								
Cryptitis, %	67	67	61	75	22	0.74	0.95	0.74
Crypt abscess, %	67	50	100	83	21	0.40	0.11	1.00
Erosion/Ulceration, %	0 ^b^	0 ^b^	37 ^a^	33 ^a^	16	0.90	0.03	0.90
Inflammation score	0.75 ^b^	0.58 ^b^	1.9 ^a^	1.9 ^a^	0.4	0.86	<0.01	0.76
Co50								
Cryptitis, %	67 ^ab^	33 ^b^	63 ^ab^	100 ^a^	19	0.90	0.08	0.05
Crypt abscess, %	83	83	100	100	14	0.97	0.22	0.97
Erosion/Ulceration, %	0 ^c^	0 ^c^	40 ^b^	83 ^a^	13	0.10	<0.001	0.10
Inflammation, score	0.75 ^c^	0.50 ^c^	1.9 ^b^	2.7 ^a^	0.2	0.18	<0.001	0.01
Co75								
Cryptitis, %	33	50	74	83	20	0.49	0.06	0.84
Crypt abscess, %	50 ^b^	67 ^b^	94 ^a^	100 ^a^	14	0.39	<0.01	0.70
Erosion/Ulceration, %	0 ^c^	0 ^c^	40 ^b^	83 ^a^	13	0.10	<0.001	0.10
Inflammation, score	0.50 ^c^	0.58 ^c^	1.7 ^b^	2.5 ^a^	0.3	0.06	<0.001	0.13

^a,b,c^ Means within a row with unlike superscript letters were significantly different (*p* ≤ 0.05).

## Supplementary Materials

The following are available online at https://www.mdpi.com/2072-6643/12/6/1728/s1, Table S1: Oligonucleotide sequences of forward primer, probe (in case it was used) and reverse primer for the studied genes.

## References

[1] Granlund A.V.B., Flatberg A., Østvik A.E., Drozdov I., Gustafsson B.I., Kidd M., Beisvag V., Torp S.H., Waldum H.L., Martinsen T.C. (2013). Whole genome gene expression meta-analysis of inflammatory bowel disease colon mucosa demonstrates lack of major differences between Crohn’s disease and ulcerative colitis. PLoS ONE.

[2] Sartor R.B. (2006). Mechanisms of disease: Pathogenesis of Crohn’s disease and ulcerative colitis. Nat. Clin. Pract. Gastroenterol. Hepatol..

[3] Ford A.C., Moayyedi P., Hanauer S.B. (2013). Ulcerative colitis. Br. Med. J..

[4] Ananthakrishnan A.N., Bernstein C.N., Iliopoulos D., Macpherson A., Neurath M.F., Ali R.A.R., Vavricka S.R., Fiocchi C. (2017). Environmental triggers in IBD: A review of progress and evidence. Nat. Clin. Pract. Gastroenterol. Hepatol..

[5] Andersen V., Olsen A., Carbonnel F., Tjønneland A., Vogel U. (2012). Diet and risk of inflammatory bowel disease. Dig. Liver Dis..

[6] Burisch J., Jess T., Martinato M., Lakatos P.L. (2013). The burden of inflammatory bowel disease in Europe. J. Crohns Colitis.

[7] Ng S.C., Shi H.Y., Hamidi N., Underwood F.E., Tang W., Benchimol E.I., Panaccione R., Ghosh S., Wu J.C.Y., Chan F.K.L. (2017). Worldwide incidence and prevalence of inflammatory bowel disease in the 21st century: A systematic review of population-based studies. Lancet.

[8] Kaplan G.G. (2015). The global burden of IBD: From 2015 to 2025. Nat. Rev. Gastroenterol. Hepatol..

[9] D’Souza S., Levy E., Mack D., Israel D., Lambrette P., Ghadirian P., Deslandres C., Morgan K., Seidman E.G., Amre D.K. (2007). Dietary patterns and risk for Crohn’s disease in children. Inflamm. Bowel Dis..

[10] Cohen A.B., Lee D., Long M.D., Kappelman M.D., Martin C.F., Sandler R.S., Lewis J.D. (2013). Dietary patterns and self-reported associations of diet with symptoms of inflammatory bowel disease. Dig. Dis. Sci..

[11] Jantchou P., Morois S., Clavel-Chapelon F., Boutron-Ruault M.-C., Carbonnel F. (2010). Animal protein intake and risk of inflammatory bowel disease: The e3n prospective study. Am. J. Gastroenterol..

[12] Pieczyńska J., Prescha A., Zabłocka-Słowińska K., Neubauer K., Smereka A., Grajeta H., Biernat J., Paradowski L. (2018). Occurrence of dietary risk factors in inflammatory bowel disease: Influence on the nutritional status of patients in clinical remission. Adv. Clin. Exp. Med..

[13] Jowett S.L., Seal C.J., Pearce M.S., Phillips E., Gregory W., Barton J.R., Welfare M.R. (2004). Influence of dietary factors on the clinical course of ulcerative colitis: A prospective cohort study. Gut.

[14] Sakamoto N., Kono S., Wakai K., Fukuda Y., Satomi M., Shimoyama T., Inaba Y., Miyake Y., Sasaki S., Okamoto K. (2005). Dietary risk factors for inflammatory bowel disease: A multicenter case-control study in japan. Inflamm. Bowel Dis..

[15] Ge J., Han T.J., Liu J., Li J.S., Zhang X.H., Wang Y., Li Q.Y., Zhu Q., Yang C.M. (2015). Meat intake and risk of inflammatory bowel disease: A meta-analysis. Turk. J. Gastroenterol..

[16] Gilbert M.S., Ijssennagger N., Kies A.K., van Mil S.W.C. (2018). Protein fermentation in the gut; implications for intestinal dysfunction in humans, pigs, and poultry. Am. J. Physiol. Gastroint. Liver Physiol..

[17] Rasmussen N.F., Rubin K.H., Stougaard M., Tjønneland A., Stenager E., Lund Hetland M., Glintborg B., Bygum A., Andersen V. (2019). Impact of red meat, processed meat and fibre intake on risk of late-onset chronic inflammatory diseases: Prospective cohort study on lifestyle factors using the Danish ‘diet, cancer and health’ cohort (PROCID-DCH): Protocol. BMJ Open.

[18] Ijssennagger N., van der Meer R., van Mil S.W.C. (2016). Sulfide as a mucus barrier-breaker in inflammatory bowel disease?. Trends Mol. Med..

[19] Maloy K.J., Powrie F. (2011). Intestinal homeostasis and its breakdown in inflammatory bowel disease. Nature.

[20] Hou J.K., Abraham B., El-Serag H. (2011). Dietary intake and risk of developing inflammatory bowel disease: A systematic review of the literature. Am. J. Gastroenterol..

[21] Kawada M.A.A., Mizoguchi E. (2007). Insights from advances in research of chemically induced experimental models of human inflammatory bowel disease. World J. Gastroenterol..

[22] Eichele D.D., Kharbanda K.K. (2017). Dextran sodium sulfate colitis murine model: An indispensable tool for advancing our understanding of inflammatory bowel diseases pathogenesis. World J. Gastroenterol..

[23] Spurlock M.E., Gabler N.K. (2008). The development of porcine models of obesity and the metabolic syndrome. J. Nutr..

[24] Liu Y., Wang X., Hou Y., Yin Y., Qiu Y., Wu G., Hu C.-A.A. (2017). Roles of amino acids in preventing and treating intestinal diseases: Recent studies with pig models. Amino Acids.

[25] Clouard C., Meunier-Salaün M.C., Val-Laillet D. (2012). Food preferences and aversions in human health and nutrition: How can pigs help the biomedical research?. Animal.

[26] Miller E.R., Ullrey D.E. (1987). The pig as a model for human nutrition. Annu. Rev. Nutr..

[27] Verma N., Rettenmeier A.W., Schmitz-Spanke S. (2011). Recent advances in the use of sus scrofa (pig) as a model system for proteomic studies. Proteomics.

[28] Kararli T.T. (1995). Comparison of the gastrointestinal anatomy, physiology, and biochemistry of humans and commonly used laboratory animals. Biopharm. Drug Dispos..

[29] Kim C.J., Kovacs-Nolan J.A., Yang C., Archbold T., Fan M.Z., Mine Y. (2010). L-tryptophan exhibits therapeutic function in a porcine model of dextran sodium sulfate (DSS)-induced colitis. J. Nutr. Biochem..

[30] Kim C.J., Kovacs-Nolan J., Yang C., Archbold T., Fan M.Z., Mine Y. (2009). L-cysteine supplementation attenuates local inflammation and restores gut homeostasis in a porcine model of colitis. Biochim. Biophys. Acta.

[31] Bassaganya-Riera J., Hontecillas R. (2006). CLA and n-3 PUFA differentially modulate clinical activity and colonic ppar-responsive gene expression in a pig model of experimental IBD. Clin. Nutr..

[32] Ibuki M., Fukui K., Kanatani H., Mine Y. (2014). Anti-inflammatory effects of mannanase-hydrolyzed copra meal in a porcine model of colitis. J. Vet. Med. Sci..

[33] Young D., Ibuki M., Nakamori T., Fan M., Mine Y. (2012). Soy-derived di- and tripeptides alleviate colon and ileum inflammation in pigs with dextran sodium sulfate-induced colitis. J. Nutr..

[34] Lackeyram D., Young D., Kim C.J., Yang C., Archbold T.L., Mine Y., Fan M.Z. (2017). Interleukin-10 is differentially expressed in the small intestine and the colon experiencing chronic inflammation and ulcerative colitis induced by dextran sodium sulfate in young pigs. Physiol. Res..

[35] Pedersen K.S., Toft N. (2011). Intra- and inter-observer agreement when using a descriptive classification scale for clinical assessment of faecal consistency in growing pigs. Prev. Vet. Med..

[36] Larsen T., Fernández C. (2017). Enzymatic-fluorometric analyses for glutamine, glutamate and free amino groups in protein-free plasma and milk. J. Dairy Res..

[37] Keshteli A.H., Madsen K.L., Dieleman L.A. (2019). Diet in the pathogenesis and management of ulcerative colitis; a review of randomized controlled dietary interventions. Nutrients.

[38] Khalili H., Chan S.S.M., Lochhead P., Ananthakrishnan A.N., Hart A.R., Chan A.T. (2018). The role of diet in the aetiopathogenesis of inflammatory bowel disease. Nat. Rev. Gastroenterol. Hepatol..

[39] O’Shea C.J., O’Doherty J.V., Callanan J.J., Doyle D., Thornton K., Sweeney T. (2016). The effect of algal polysaccharides laminarin and fucoidan on colonic pathology, cytokine gene expression and enterobacteriaceae in a dextran sodium sulfate-challenged porcine model. J. Nutr. Sci..

[40] Ritchie L.E., Taddeo S.S., Weeks B.R., Carroll R.J., Dykes L., Rooney L.W., Turner N.D. (2017). Impact of novel sorghum bran diets on DSS-induced colitis. Nutrients.

[41] Panasevich M.R., Allen J.M., Wallig M.A., Woods J.A., Dilger R.N. (2015). Moderately fermentable potato fiber attenuates signs and inflammation associated with experimental colitis in mice. J. Nutr..

[42] Pistol G.C., Marin D.E., Rotar M.C., Ropota M., Taranu I. (2020). Bioactive compounds from dietary whole grape seed meal improved colonic inflammation via inhibition of mapks and nf-kb signaling in pigs with DSS-induced colitis. J. Funct. Foods.

[43] Matsuda R., Koide T., Tokoro C., Yamamoto T., Godai T., Morohashi T., Fujita Y., Takahashi D., Kawana I., Suzuki S. (2009). Quantitive cytokine mRNA expression profiles in the colonic mucosa of patients with steroid naive ulcerative colitis during active and quiescent disease. Inflamm. Bowel Dis..

[44] Del Valle-Pinero A.Y., Van Deventer H.E., Fourie N.H., Martino A.C., Patel N.S., Remaley A.T., Henderson W.A. (2013). Gastrointestinal permeability in patients with irritable bowel syndrome assessed using a four probe permeability solution. Clin. Chim. Acta.

[45] Schepens M.A., Vink C., Schonewille A.J., Dijkstra G., van der Meer R., Bovee-Oudenhoven I.M. (2011). Dietary heme adversely affects experimental colitis in rats, despite heat-shock protein induction. Nutrition.

[46] Otaka M., Odashima M., Watanabe S. (2006). Role of heat shock proteins (molecular chaperones) in intestinal mucosal protection. Biochem. Biophys. Res. Com..

[47] Manichanh C., Borruel N., Casellas F., Guarner F. (2012). The gut microbiota in IBD. Nature Rev. Gastroenterol. Hepatol..

[48] Kostic A.D., Xavier R.J., Gevers D. (2014). The microbiome in inflammatory bowel disease: Current status and the future ahead. Gastroenterol..

[49] Morgan X.C., Tickle T.L., Sokol H., Gevers D., Devaney K.L., Ward D.V., Reyes J.A., Shah S.A., LeLeiko N., Snapper S.B. (2012). Dysfunction of the intestinal microbiome in inflammatory bowel disease and treatment. Gen. Biol..

