# Supplementary material for: Dietary Red Meat Adversely Affects Disease Severity in a Pig Model of DSS-Induced Colitis Despite Reduction in Colonic Pro-Inflammatory Gene Expression
Source: Nutrients. 2020 Jun 9;12(6):1728. doi: 10.3390/nu12061728 (PMC7353045; doi:10.3390/nu12061728)
Supplement: Supplementary file 1 [file nutrients-12-01728-s001.pdf]

Suppl. Table 1. Oligonucleotide sequences of forward primer, probe (in case it was used) and reverse primer for the studied genes

Gene symbol	Method	Assay ID. TaqMan, Life Technologies	Forward Primer	Probe	Reverse Primer
PTSG2	TaqMan	Ss03394694_mL			
IL17A	TaqMan	Ss03391803_mL			
TGFβ	TaqMan	Ss03382325_uL			
IL6	TaqMan		5'-tcctcggcaaaatctctgcaat	FAM-5'-ttctcacacatctcctttctc	5'-tccttgctgttttcacacttctcat
IL8	TaqMan		5'-tcctgctttctgcagctctct	FAM-5'-tgaggctgcagttct	5'-catcgaagttctgcacttactcttg
IL10	TaqMan		5'-gaggaggtgaagagtgccttta	FAM-5'-cctctcttggagcttgc	5'-ctcacccatggctttgtagaca
TNFα	TaqMan		5'-aaccctctggcccaagga	FAM-5'-tcagatcatcgtctcaaac	5'-ggcgacgggcttatctga
NFκβ	TaqMan		5'-ctagtgaaccgaaaccttttctctactat	FAM-5'-ctgaaatcaaagataaagagg	5'-gcttctgccgtttcctttgt
β-actin	TaqMan		5'-tccagaggcgctcttcca	VIC-5'-tcctgggcatggagt	5'-cgcacttcatgatcgagttga
GAPDH	TaqMan		5'- gtcggagtgaacggatttgg	5'-cgcctggtcaccagggctgct	5'-caatgtccactttgccagagttaa
